# The theme of the world diabetes day 2014; healthy living and diabetes; a nephrology viewpoint

**Published:** 2014-07-01

**Authors:** Seyed Seifollah Beladi-Mousavi, Bahman Bashardoust, Hamid Nasri, Ali Ahmadi, Zahra Tolou-Ghamari, Shabnam Hajian, Sara Torkamaneh

**Affiliations:** ^1^Chronic Renal Failure Research Center, Department of Internal Medicine, Ahwaz Jundishapur University of Medical Sciences, Ahvaz, Iran; ^2^Department of Internal Medicine, Imam Khomeini Hospital, Ardabil University of Medical Sciences, Ardabil, Iran; ^3^Department of Nephrology, School of Medicine, Isfahan University of Medical Sciences, Isfahan, Iran; ^4^Department of Epidemiology and Biostatistics, School of Public Health, Shahrekord University of Medical Sciences, Shahrekord, Iran; ^5^Isfahan Neurosciences Research Centre, Faculty of Medicine, Isfahan University of Medical Sciences, Isfahan, Iran; ^6^Nickan Research Institute, Isfahan, Iran; ^7^Department of Physical Education and Sport Science, Khorasgan University, Isfahan, Iran

**Keywords:** Type II diabetes, World diabetes day, β-cell dysfunction

## Abstract

Annually, on November 14, the world diabetes day (WDD) is celebrated. WDD is a campaign led by the International Diabetes Federation (IDF) and its member associations throughout the world. It was created in 1991 by IDF and World Health Organization (WHO) in response to increasing concerns about the intensifying threat of diabetes worldwide. The WDD 2014 organization marks the first of a three-year (2014-16) emphasis on "healthy living and diabetes". Replacement of whole grain and cereal-based foods with refined grains in diet planning could be an operative and practical strategy in type II diabetic patients. This strategy beyond the development of glycemic control, leads to more benefits for management of other features of diabetes, diminution of diabetes-induced metabolic disorders, and prevents long-term complications especially diabetic kidney disease and cardiovascular disease.

**Implication for health policy/practice/research/medical education:**

Annually, on November 14, the world diabetes day (WDD) is celebrated. WDD is a campaign led by the International Diabetes Federation (IDF) and its member associations throughout the world. It was created in 1991 by IDF and WHO in response to increasing concerns about the intensifying threat of diabetes worldwide. The WDD 2014 organization marks the first of a three-year (2014-16) emphasis on "healthy living and diabetes".

## Introduction

Annually, on November 14, the world diabetes day (WDD) is celebrated. WDD is a campaign led by the International Diabetes Federation (IDF) and its member associations throughout the world. It was created in 1991 by IDF and World Health Organization (WHO) in response to increasing concerns about the intensifying threat of diabetes worldwide. The WDD 2014 organization marks the first of a three-year (2014-16) emphasis on “healthy living and diabetes”. This activities and materials in these years will particularly concentrate on the subject of healthy eating and its significance both in the prevention of type II diabetes and the appropriate management of diabetes to prevent complications (1-3). Type II diabetes mellitus is a metabolic disease defined by developing insulin resistance, β-cell dysfunction, impaired insulin secretion and finally hyperglycemia (1-3). Type II diabetes mellitus is a complicated metabolic disease with equally short- and long-term undesirable problems. In fact, diabetes mellitus is a main non-communicable illness and presently, an important communal health problem, while the prevalence of diabetes mellitus is growing and the percentage of individuals with the disease will double by 2025. An additional 316 million people are presently at high risk of obtaining type II diabetes, with the number anticipated to increase to almost 500 million within a generation (1-3).

## Oxidative stress in diabetes

Various metabolic disorders comprising impaired lipid metabolism, oxidative stress, micro-inflammation, vascular endothelial cell dysfunction and high blood pressure are commonly accompanied by type II diabetes mellitus. During the past two decades, the concept of antioxidant therapy in diabetes mellitus is fast expanding (4,5). Oxidative stress is a condition with over production of free radicals and defect in endogenous antioxidant defense system (4,5). Antioxidant therapy is also a main part of type II diabetes mellitus management (4-6). Foods containing antioxidant, beyond the basic nutritional functions have potential profits to promote health and diminish the risk of chronic diseases and have hence been given much concentration (5-7). In recent years, investigations have centered on properties of the bioactive compounds of antioxidant foods in the control of some features of diabetes mellitus. Oxidative stress is believed as imbalance between the reactive oxygen species (ROS) and antioxidants system in scavenging the reactive intermediates (6-8). Oxidation reactions are crucial for living, however, they can also be damaging (5-9). Oxidation is a chemical reaction which usually transfers electrons from a substance to an oxidizing agent (7-9). Insufficient levels of antioxidants, cause oxidative stress which may injure the components of the cells, containing deoxyribonucleic acids, proteins and lipids (5-9). The oxidative stress is implicated in the development of atherosclerosis, myocardial infarction, heart failure, Parkinson’s disease, Alzheimer’s disease and kidney insufficiency (8,9). Antioxidants are reducing agents like ascorbic acid and polyphenols molecules that inhibit the oxidation of other molecules. Animals maintain complex systems of multiple types of antioxidants such as vitamin A, C and E, glutathione, and also enzymes such as catalase, superoxide dismutase and various peroxidases (5-10). Antioxidants are extensively used in dietary supplements and have been investigated for the prevention of diseases such as cancer, coronary heart disease and even altitude sickness. Various investigations have shown beneficial effects with antioxidant supplements, however, large clinical trials have shown no benefit and even in some cases suggested that excess supplementation with certain putative antioxidants may be harmful (6-10). Indeed, fruits and vegetables are nearly almost beneficial, however this is not the case for diet supplementations. The exact mechanism is not well-defined, however the possible description is that, in the fruits and vegetables, there is a mix of antioxidants and they act as a continuous chain. Additionally, antioxidant supplementation is usually given using one or two substances. Hence, the antioxidant chain is not completely available (6-10). In this regard, after scavenging free radicals, if an antioxidant is not restored by the following antioxidant in the chain, it begins to be a pro-oxidant. The final impact of such supplementations, in this situation, would be no effect or a damaging effect (5-9). Thus, in antioxidant therapy complimentary antioxidants cannot always substitute the fruits and vegetables high in antioxidants. On the other hand, one of the main aspects of healthy living and foods in diabetes is the quality and quantity and quality of the nutrition during pregnancy might cause strong and permanent consequences on the fetus. The distorted structure of chromosome during this procedure may be the cause of cell dysfunction and increased predisposition to diseases throughout altered gene expression (6-10).

## The theme of healthy living and diabetes in 2014

It is well defined that, the relationships between maternal malnutrition, low protein diet, and type II diabetes mellitus have been widely investigated. In fact, estimation of energy and nutrients necessities, carbohydrate counting and glycemic index and also glycemic load, suggestion for dietary fats and cholesterol and protein intakes, as well as using natural safe antioxidants are the common important suggestions for a healthy diet for a pregnant woman with diabetes (11-14). What makes the pandemic especially menacing is that throughout much of the world, it persists hidden. Indeed, up to half of all individuals with DM globally remain undiagnosed. These data and aspects restate the importance of urgent action. The majority cases of type II diabetes can be prevented and the serious complications of diabetes can be prevented through healthy lifestyles and living environments which encourage and facilitate healthy behavior (12-16). The main messages of WDD sought to raise alertness of how the healthy choice can be the easy choice and the various steps that persons can take to make informed decisions about what they eat. No food is out of limits but food choices are an important part of the diabetes management. Consuming a balanced diet – that is vegetables and fruit, starchy foods, non-dairy sources of protein and dairy – is something we should for diabetic individuals (11-16). It should be remember that, foods, we recommend are an important part of the diabetes treatment, along with their medication. This evidence is a starting point to help the diabetics eat well when they have diabetes. However, diabetics should also be referred to a registered dietitian for specific knowledge tailored to their needs. A particular focus however, will be put toward the importance of starting the day with a healthy breakfast (12-17). Bread, potatoes, rice and pasta contain carbohydrate, which is broken down into glucose and utilized by cells. Choose carbohydrates that are more gradually absorbed as these carbohydrates will not affect the blood glucose levels as much and they will keep you feeling fuller for longer. Additionally, starchy foods are naturally low in fat and high-fiber choices will also keep the bowel regular, preventing digestive disorders. Vegetables and Fruits are naturally low in calories and fat and, while being stored with vitamins, minerals and fiber and also antioxidants. They can help protect against high blood pressure, stroke, some cancers and heart disease. Likewise, milk, yoghurt and cheese contain calcium, which helps to keep the bones and teeth strong. They are a good source of protein too (14-17). Additionally, meat, eggs and fish, are high in protein, which is demanded for building and replacing muscle cells in the body. They also comprise minerals, such as iron, which are demanded for producing red blood cells. Omega-3 fish oils, located in oily fish such as mackerel, salmon and sardines, can help to save the heart (11-17). In summary, type II diabetes mellitus is a multifactorial disorder, and its etiology involves a complex interaction between genetic, epigenetic, and environmental factors. Since the incidence of DM is increasing global at an alarming rate, hence an appropriate understanding of the mechanisms and effective treatment of the disease is becoming gradually important. One of the mostly important issue near diabetics, is healthy living, which starts with a healthy eating. This is a theme of WDD 2014 to emphasis more cooperation of dietitian, endocrinologist and finally nephrologists to avoid diabetic complications (13-20).

## Conclusion

In conclusion, replacement of whole grain and cereal-based foods with refined grains in diet planning could be an operative and practical strategy in type II diabetic patients. This strategy beyond the development of glycemic control, leads to more benefits for management of other features of diabetes, diminution of diabetes-induced metabolic disorders, and prevents long-term complications especially diabetic kidney disease and cardiovascular disease.

## Authors’ contributions

All authors contributed to the paper equally.

## Conflict of interests

The authors declared no competing interests.

## Ethical considerations

Ethical issues (including plagiarism, misconduct, data fabrication, falsification, double publication or submission, redundancy) have been completely observed by the authors.

## Funding/Support

None.
